# Real-world safety profile and mechanistic insights into regorafenib-induced liver failure: a pharmacovigilance study integrated with network toxicology

**DOI:** 10.3389/fphar.2025.1698511

**Published:** 2026-01-12

**Authors:** Haonan Sun, Heng Gu, Pengkai Xu, Dong Jiang, Tian Pu, Jiangming Chen, Fubao Liu

**Affiliations:** Department of Hepatobiliary Surgery, First Affiliated Hospital of Anhui Medical University, Hefei, China

**Keywords:** FAERS, liverfailure, network toxicology, pharmacovigilance, regorafenib

## Abstract

**Background:**

Regorafenib is a multikinase inhibitor widely used in oncology, but it is associated with significant adverse events (AEs), particularly hepatotoxicity. Understanding the real-world safety profile and the molecular mechanisms underlying regorafenib-induced liver failure is critical for clinical risk management.

**Methods:**

AE reports with regorafenib as the primary suspect drug were extracted from the FDA Adverse Event Reporting System (FAERS) from Q3 2012 to Q1 2025. Disproportionality analyses were conducted using reporting odds ratio (ROR), proportional reporting ratio (PRR), multi-item gamma Poisson shrinker (MGPS), and Bayesian confidence propagation neural network (BCPNN). Clinical prioritization was assessed via a semi-quantitative framework. Time-to-onset was analyzed using Weibull distribution. Additionally, network toxicology and molecular docking were employed to explore potential mechanisms and binding affinities between regorafenib and liver failure targets.

**Results:**

A total of 9,442 AE reports were analyzed. Hepatobiliary disorders exhibited the strongest signal strength (ROR = 2.66, 95% CI: 2.50–2.83), with hepatic failure and fulminant hepatitis identified as high-priority AEs. The majority of AEs occurred within 30 days of treatment initiation. Subgroup analysis indicated that elderly patients (≥65 years) and males had higher risks of liver failure, whereas higher body weight appeared protective. Network analysis identified 63 overlapping targets, with MAPK and PI3K-Akt signaling pathways significantly enriched.

**Conclusion:**

This pharmacovigilance analysis identifies regorafenib-associated hepatotoxicity as a significant safety signal, with higher risks observed in elderly and male patients. Computational predictions suggest the involvement of MAPK and PI3K-Akt pathways, offering a theoretical basis for further mechanistic investigation and supporting the need for vigilant clinical monitoring.

## 1 Introduction

Regorafenib is an oral multikinase inhibitor targeting protein kinases involved in angiogenesis, oncogenesis, metastasis, and tumor microenvironment modulation. Specifically, it inhibits a spectrum of receptors, including vascular endothelial growth factor receptors (VEGFR1, VEGFR2, and VEGFR3), TIE2 (a critical regulator of angiogenesis), KIT, RAF1, RET, BRAF (V600E mutant), platelet-derived growth factor receptors (PDGFRs), and fibroblast growth factor receptors (FGFRs) (Tsai et al., 2021). Regorafenib was initially approved for previously treated metastatic colorectal cancer and gastrointestinal stromal tumors, and later for hepatocellular carcinoma progressing after sorafenib (Bruix et al., 2017; Ciracì et al., 2025; Dedousis et al., 2025). Furthermore, regorafenib is under active investigation for treating various solid tumors, including cholangiocarcinoma, osteosarcoma, soft-tissue sarcoma, and neuroblastoma (Demols et al., 2020; Han et al., 2025; Toulmonde et al., 2025; Zeng et al., 2023).

Regorafenib has been extensively employed in clinical practice and demonstrated efficacy. Given its widespread clinical use, evaluating the real-world safety profile of regorafenib is critically important. Current data on regorafenib-associated adverse events predominantly derive from short-term clinical trials; however, these studies typically enroll specific patient populations under strict inclusion criteria with limited sample sizes, thus limiting comprehensive assessment of the drug’s adverse reactions. The prescribing information indicates that the most frequently reported adverse events (AEs) associated with regorafenib include pain, hand-foot skin reaction, fatigue, and diarrhea. Of particular concern is regorafenib-induced severe hepatotoxicity, for which the FDA has issued a black box warning. Severe toxic hepatitis occurred in some patients receiving regorafenib, and a significant proportion developed acute liver failure during treatment.

The FDA Adverse Event Reporting System (FAERS) is a publicly accessible database comprising extensive records of adverse drug reactions (Alatawi and Hansen, 2017). As the largest global pharmacovigilance database, it serves as a valuable resource for identifying drug-related adverse events. To further evaluate the real-world safety profile of regorafenib, we aim to analyze regorafenib-associated AEs documented within FAERS, thereby providing supplementary evidence to inform clinical practice. Understanding the intrinsic biological mechanisms underlying regorafenib-induced liver failure is critical for risk prediction, early diagnosis, and the development of preventive strategies. However, this mechanism remains elusive. Network pharmacology, an emerging bioinformatics methodology, enables systematic elucidation of drug toxicity mechanisms (Wang et al., 2023). Therefore, network pharmacology approaches were employed to predict and identify key molecular targets and signaling pathways potentially mediated by regorafenib in liver failure, and to preliminarily elucidate its potential molecular mechanisms.

## 2 Materials and methods

### 2.1 Data sources and processing

This study extracted AE reports associated with regorafenib (including its trade name, STIVARGA) from the FAERS database, covering the period from the third quarter of 2012 through the first quarter of 2025. Reports were included only if regorafenib was designated as the primary suspect (PS) drug. Data extraction and cleansing were performed using R software (version 4.4.3). The initial database comprised 19,092,746 reports. Reports were deemed duplicates if they shared identical key identifiers, including the patient’s unique identifier (when available), the date of the AE, the reported drug, and the description of the AE. When multiple reports involved the same patient and drug-related AE, only the most recent entry was retained. This approach ensured that each unique AE instance was counted only once, thereby reducing the potential over-representation of specific events in the analysis. After deduplication, 16,426,538 unique drug-related reports remained for subsequent analysis. The dataset encompassed demographic information, concomitant drugs, indications, reported adverse reactions, and patient outcomes. For demographic variables such as sex, age, and body weight, missing data were retained and reported as distinct categories in the descriptive clinical characteristics analysis. AEs were coded using the Medical Dictionary for Regulatory Activities (MedDRA, version 27.0) at the Preferred Term (PT) level. Subsequently, PTs were categorized according to their respective System Organ Classes (SOCs).

### 2.2 Data mining

To assess significant associations between regorafenib and AEs, we utilized multiple pharmacovigilance methodologies: specifically, the reporting odds ratio (ROR), proportional reporting ratio (PRR), multi-item gamma Poisson shrinker (MGPS), and Bayesian confidence propagation neural network (BCPNN). The integration of these diverse methods is crucial for robust signal detection, as each offers unique strengths. ROR and PRR are traditional frequentist methods, effective for identifying common signals and easy to interpret. In contrast, the Bayesian methodologies (MGPS, BCPNN) are more robust for identifying rare safety signals and less susceptible to data sparsity, thus providing greater stability and reliability. The Bayesian methodologies (MGPS, BCPNN) demonstrated enhanced robustness in identifying rare safety signals. The Bayesian methodologies (MGPS, BCPNN) demonstrated enhanced robustness in identifying rare safety signals. To minimize the detection of false positive signals, potential signals were defined as AEs exceeding the positivity threshold in at least one of these methodologies. This inclusive approach ensures comprehensive signal capture, leveraging the strengths of each method to identify any disproportionately reported AE. The two-by-two contingency table and detailed formulas for these methods of disproportionality analysis and the widely accepted positive signal thresholds are provided in Supplementary Table S1.

### 2.3 Clinical prioritization evaluation

The primary objective of clinical priority assessment is to identify signals indicative of significant risks to patient or public health, or those with the potential to substantially alter the risk-benefit profile of a medicinal product, thereby necessitating urgent mitigation. A semi-quantitative assessment of emerging signals within the PT tier was conducted, evaluating each signal across four key dimensions: adherence to Important Medical Event (IME) or Designated Medical Event (DME) criteria, reporting rate, signal stability, and reported case fatality rate (Table 1). IMEs (serious events—version 26.0) and DMEs (rare but serious events with a high likelihood of drug causation) are established and standardized definitions issued by the European Medicines Agency. Each dimension was stratified into three levels, assigned scores of 0, 1, or 2 respectively. AEs were subsequently categorized as low, moderate, or high clinical priority based on cumulative scores falling within the ranges of 0–2, 3–5, or 6–8, respectively (Cecco et al., 2024).

**TABLE 1:** Criteria and relevant scores to prioritize AEs emerged from disproportionality analysis.

Criterium	2 points	1 point	0 point
Reporting rate (cases/non-cases)	>10%	1%–10%	0%–1%
Signal stability (consistency across disproportionality analyses)	3 of 3	2 of 3	1 of 3
Reported case fatality rate (proportion of reports with death as outcome)	>50%	25%–50%	<25%
DMEs or IMEs	DME	IME	None

AEs, adverse events; DME, designated medical event; IME, important medical event.

### 2.4 Time-to-onset analysis

The time-to-onset (TTO) of an AE was defined as the interval between the start date of regorafenib treatment and the reported AE onset date. Only reports with complete information on both the start date of regorafenib treatment and the AE onset date were included to ensure accurate calculation of TTO, leading to the exclusion of cases with missing onset dates. Onset times were summarized using median values and interquartile ranges (IQR). Temporal patterns in AE incidence were examined using Weibull distribution analysis. The Weibull distribution is characterized by two parameters: scale (α) and shape (β). The shape parameter (β) of the Weibull distribution characterizes the underlying hazard function for AE occurrence over time: If β < 1, the AE is mainly concentrated in the early stage of administration of the medication (early failure-type profile); if β = 1, the AE occurs randomly with no obvious time aggregation (random failure-type profile); and β > 1 is thought to indicate an increase with time (wear-out failure-type profile) (Sauzet and Cornelius, 2022). Furthermore, cumulative AE incidences were visualized using Kaplan-Meier curves, with between-group comparisons performed using the log-rank test. P < 0.05 was considered statistically significant. All statistical analyses were performed in R software (version 4.2.3; R Foundation, Vienna, Austria).

### 2.5 Network toxicological analysis

In this study, predicted targets of regorafenib were obtained from the STITCH and ChEMBL databases. Relevant targets of key AEs were collected from the GeneCards and OMIM databases. Subsequently, a Venn diagram was utilized to identify the intersection between the regorafenib-predicted targets and the AE-associated targets; these overlapping targets were defined as the candidate toxicity targets associated with regorafenib-induced AEs. A protein-protein interaction (PPI) network for these putative toxicity targets was constructed using the STRING database. For the identification of core targets, network topology analysis was performed, with degree centrality as the primary evaluation indicator. The screening criterion for core targets was defined by selecting the top 10 nodes with the highest degree centrality scores within this PPI network, as these represent the most highly connected nodes and key regulatory hubs. The PPI network was visualized using Cytoscape software (version 3.8.2) (Szklarczyk et al., 2023). Key targets within this network were subsequently identified based on degree. Gene ontology (GO) functional analysis and Kyoto Encyclopedia of Genes and Genomes (KEGG) pathway enrichment analysis of potential toxicity targets for key AEs were performed using the DAVID database (https://david.ncifcrf.gov/) (Sherman et al., 2022) and visualized using the bioinformatics online platform (https://www.bioinformatics.com.cn/).

### 2.6 Molecular docking

Molecular docking was utilized to further elucidate the intermolecular interactions between regorafenib and the key target proteins previously identified in this study, by predicting their binding modes and affinities (Paggi et al., 2024). The X-ray crystal structures of the key proteins were retrieved from the RCSB Protein Data Bank (PDB) (https://www.rcsb.org). The 3D structure of regorafenib was obtained from PubChem (https://pubchem.ncbi.nlm.nih.gov/). Target protein structures were prepared using PyMOL (version 4.6.0) by removing water molecules and co-crystallized ligands, followed by addition of hydrogen atoms, assignment of atomic charges, and merging of non-polar hydrogens using AutoDock Tools (version 1.5.6). Molecular docking proceeded with AutoDock Vina after sizing the Grid Box and genetic algorithm. The grid box was centered to cover the entire protein structure to cover the binding pocket. Molecular docking proceeded with AutoDock Vina. The binding affinity was used as the primary evaluation indicator. A binding energy < −5.0 kcal/mol was defined as the threshold for stable binding activity. The results were visualized using Pymol.

## 3 Results

### 3.1 Clinical characteristics

**FIGURE 1:**
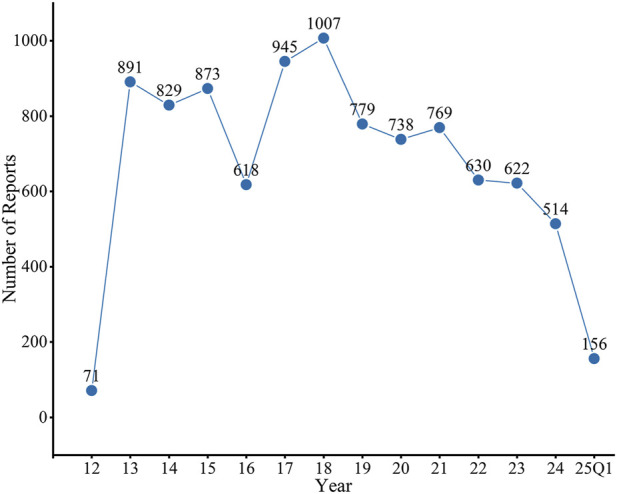
Distribution of AEs of regorafenib from the third quarter of 2012 (2012 Q3) to the first quarter of 2025 (2025 Q1). FAERS, Food and Drug Administration (FDA) Adverse Event Reporting System (FAERS); AEs, adverse events.

A total of 9,442 AE reports associated with regorafenib as the PS were included in this study. As illustrated in Figure 1, the annual number of reports increased rapidly following market entry, peaked in 2018 (n = 1,007), and subsequently exhibited fluctuations, followed by a continuous downward trend. Of these, AE reports from males accounted for 54.67%, significantly higher than those from females at 38.02%. Regarding age distribution, patients aged 18–65 years accounted for the majority of reports (n = 3,841, 40.68%), followed closely by those in the 65–85 years age group (n = 3,382, 35.16%). The principal source of reports was the United States (n = 4,682,49.59%), and physicians were the most common reporters (n = 3,040, 32.20%). Among the reported outcomes, hospitalization (n = 2,716, 28.77%) was the most common severe outcome. Colorectal cancer is the most common indication (n = 5,880,62.27%), followed by hepatic cancer (n = 957,10.14%) and gastrointestinal stromal tumor (n = 578,6.12%) (Table 2).

**TABLE 2:** Demographic characteristics of AEs reported with regorafenib.

Characteristics	Case number	Case proportion (%)
All report	9,442	100.00
Sex		
Female	3,590	38.02
Male	5,162	54.67
Missing	690	7.31
Weight (kg)		
<50	262	2.77
50–100	1,430	15.15
>100	107	1.13
Missing	7,643	80.95
Age (years)		
<18	146	1.55
18–64	3,841	40.68
65–85	3,320	35.16
>85	77	0.82
Missing	2058	21.80
Reporter		
Physician	3,040	32.20
Consumer	2,687	28.46
Pharmacist	1,121	11.87
Other health-professional	2,594	27.47
Outcomes		
Hospitalization	2,716	28.77
Death	1875	19.86
Life-threatening	205	2.17
Disability	54	0.57
Others	4,592	48.63
Reported countries (top 5)		
United States	4,682	49.59
Japan	1,416	15.00
China	583	6.17
France	493	5.22
Canada	272	2.88
Indication (top 3)		
Colorectal cancer	5,880	62.27
Hepatic cancer	957	10.14
Gastrointestinal stromal tumor	578	6.12

AEs, adverse events.

### 3.2 Signal detection

**FIGURE 2:**
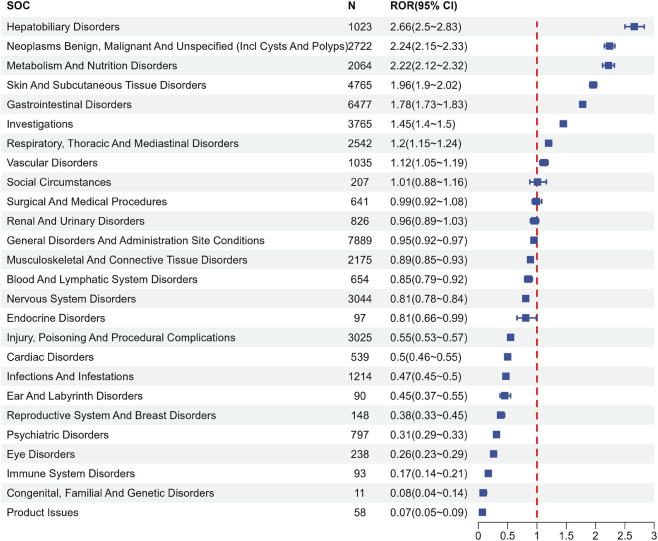
Signals detection at the SOC level. SOC, system organ class; N, number of the reports; ROR, reporting odds ratio; CI, confidence interval.

AEs associated with regorafenib were reported in 26 SOCs. Signal strengths and reports of regorafenib at the SOC level are described in Supplementary Table S2. Figure 2 and Table 3 present the signal strength (ROR and 95% CI) of regorafenib-associated AEs at the SOC level.. The most prevalent category was general disorders and administration site conditions (n = 7,889, ROR (95% CI) = 0.95 (0.92–0.97)). The SOCs that met the criteria of both algorithms include hepatobiliary disorders (n = 1,023, ROR (95% CI) = 2.66 (2.5–2.83)), neoplasms benign, malignant and unspecified (incl cysts and polyps) (n = 2,722, ROR (95% CI) = 2.24 (2.15–2.33)) and metabolism and nutrition disorders (n = 2,064, ROR (95% CI) = 2.22 (2.12–2.32)), whereas the strongest signal intensity was observed for hepatobiliary disorders.

**TABLE 3:** Signal strength of reports of regorafenib at the System Organ Class (SOC) level in FAERS database.

System Organ Class (SOC)	Case number	ROR (95% two-side CI)	PRR (χ2)	EBGM (EBGM05)	IC (IC025)
Hepatobiliary disorders	1,023	2.66 (2.50–2.83)^a^	2.62 (1,032.91)^a^	2.62 (2.49)^a^	1.39 (1.30)^a^
Neoplasms benign, malignant and unspecified (incl cysts and polyps)	2,722	2.24 (2.15–2.33)^a^	2.16 (1748.82)^a^	2.16 (2.09)^a^	1.11 (1.06)^a^
Metabolism and nutrition disorders	2064	2.22 (2.12–2.32)^a^	2.16 (1,317.12)^a^	2.16 (2.08)^a^	1.11 (1.05)^a^
Skin and subcutaneous tissue disorders	4,765	1.96 (1.90–2.02)^a^	1.86 (1999.46)	1.86 (1.81)	0.89 (0.85)^a^
Gastrointestinal disorders	6,477	1.78 (1.73–1.83)^a^	1.67 (1896.85)	1.67 (1.63)	0.74 (0.70)^a^
Investigations	3,765	1.45 (1.40–1.5)^a^	1.41 (483.13)	1.41 (1.37)	0.50 (0.45)^a^
Respiratory, thoracic and mediastinal disorders	2,542	1.2 (1.15–1.24)^a^	1.18 (76.47)	1.18 (1.15)	0.24 (0.19)^a^
Vascular disorders	1,035	1.12 (1.05–1.19)^a^	1.11 (12.04)	1.11 (1.06)	0.15 (0.06)^a^
Social circumstances	207	1.01 (0.88–1.16)	1.01 (0.04)	1.01 (0.90)	0.02 (−0.18)
Surgical and medical procedures	641	0.99 (0.92–1.08)	0.99 (0.02)	0.99 (0.93)	−0.01 (−0.12)
Renal and urinary disorders	826	0.96 (0.89–1.03)	0.96 (1.48)	0.96 (0.91)	−0.06 (−0.16)
General disorders and administration site conditions	7,889	0.95 (0.92–0.97)	0.96 (19.17)	0.96 (0.94)	−0.06 (−0.10)
Musculoskeletal and connective tissue disorders	2,175	0.89 (0.85–0.93)	0.90 (26.77)	0.90 (0.87)	−0.16 (−0.22)
Blood and lymphatic system disorders	654	0.85 (0.79–0.92)	0.85 (16.43)	0.85 (0.80)	−0.23 (−0.34)
Nervous system disorders	3,044	0.81 (0.78–0.84)	0.82 (127.03)	0.82 (0.80)	−0.28 (−0.34)
Endocrine disorders	97	0.81 (0.66–0.99)	0.81 (4.31)	0.81 (0.69)	−0.30 (−0.59)
Injury, poisoning and procedural complications	3,025	0.55 (0.53–0.57)	0.58 (1,043.8)	0.58 (0.56)	−0.79 (−0.84)
Cardiac disorders	539	0.50 (0.46–0.55)	0.51 (259.21)	0.51 (0.48)	−0.97 (−1.10)
Infections and infestations	1,214	0.47 (0.45–0.50)	0.49 (689.94)	0.49 (0.47)	−1.04 (−1.12)
Ear and labyrinth disorders	90	0.45 (0.37–0.55)	0.45 (60.61)	0.45 (0.38)	−1.15 (−1.45)
Reproductive system and breast disorders	148	0.38 (0.33–0.45)	0.39 (145.29)	0.39 (0.34)	−1.37 (−1.61)
Psychiatric disorders	797	0.31 (0.29–0.33)	0.32 (1,192.15)	0.32 (0.31)	−1.63 (−1.73)
Eye disorders	238	0.26 (0.23–0.29)	0.26 (506.03)	0.26 (0.24)	−1.93 (−2.12)
Immune system disorders	93	0.17 (0.14–0.21)	0.17 (368.55)	0.17 (0.15)	−2.52 (−2.82)
Congenital, familial and genetic disorders	11	0.08 (0.04–0.14)	0.08 (116.22)	0.08 (0.05)	−3.64 (−4.47)
Product issues	58	0.07 (0.05–0.09)	0.07 (705.71)	0.07 (0.06)	−3.79 (−4.17)

ROR, reporting odds ratio; CI, confidence interval; PRR, proportional reporting ratio, *χ2*, chi-squared; IC, information component, IC025, the lower limit of 95% CI of the IC, EBGM, empirical Bayesian geometric mean, EBGM05, the lower limit of 95% CI of EBGM.

^a^ Indicates statistically significant signals in algorithm.

**FIGURE 3:**
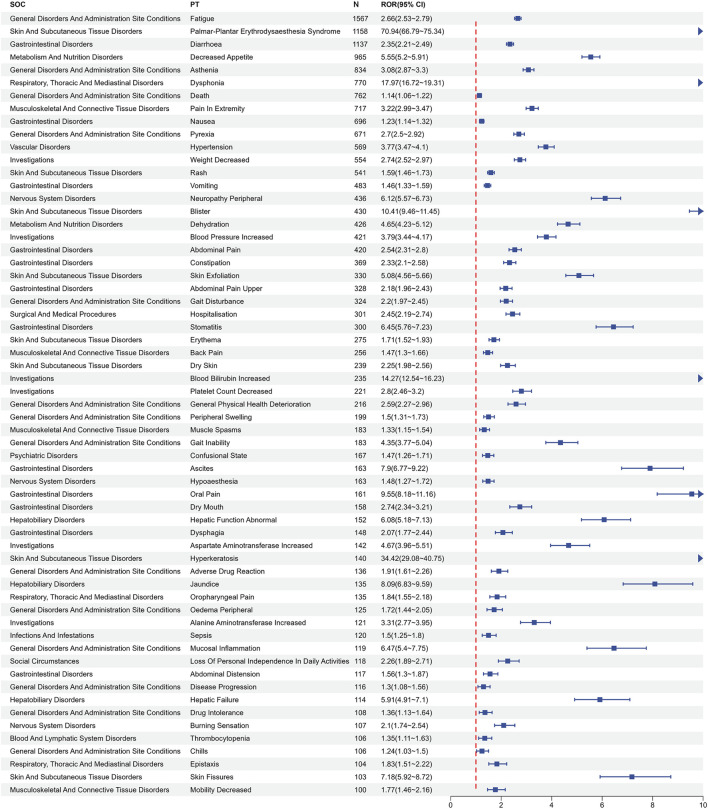
Signals detection at the PT level. PT, preferred term; CI, confidence interval; ROR, reporting odds ratio.

Following the exclusion of AEs attributable to drug characteristics or non-pharmacological factors, such as “injury, poisoning and procedural complications” and “product issues”, a total of 199 PTs satisfied all four algorithmic criteria. The ROR values of the top 50 most frequently reported PTs are listed in Figure 3. All PTs meeting the positive signal criteria were exhaustively documented in Supplementary Table S3. The five most frequently reported PTs comprised fatigue (n = 1,567, ROR (95% CI) = 2.66 (2.53 - 2.79)), palmar-plantar erythrodysaesthesia syndrome (n = 1,158, ROR (95% CI) = 70.94 (66.79–75.34)), diarrhea (n = 1,137, ROR (95% CI) = 2.35 (2.21–2.49)), decreased appetite (n = 965, ROR (95% CI) = 5.55 (5.20–5.91)) and asthenia (n = 834, ROR (95% CI) = 3.08 (2.87–3.30)). The top five PTs ranked by ROR were oral hyperaesthesia (n = 5, ROR (95%CI) = 174.49 (67.7–449.75)), thermohyperaesthesia (n = 5, ROR (95%CI) = 168.86 (65.66–434.3)), plantar erythema (n = 23, ROR (95%CI) = 143.39 (92.73–221.72)), palmar-plantar erythrodysaesthesia syndrome (n = 1,158, ROR (95% CI) = 70.94 (66.79–75.34)), and food refusal (n = 31, ROR (95% CI) = 51.14 (35.66–73.35)). Notably, new potential AE signals of clinical value were identified, such as oral hyperaesthesia, gait deviation, and hyperkeratosis, among others.

### 3.3 Subgroup analysis

**FIGURE 4:**
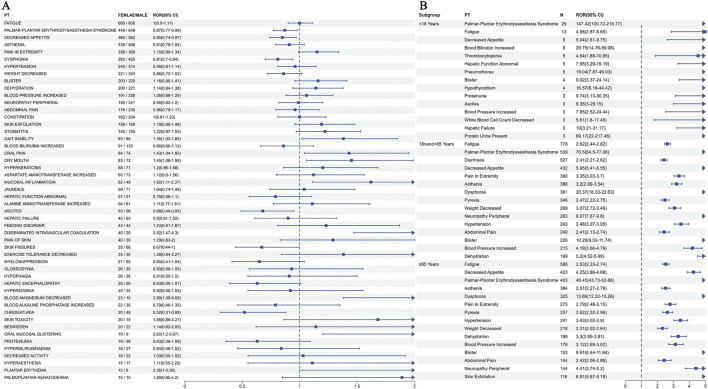
Subgroup analysis results of regorafenib adverse events. **(A)** Analysis results of the gender subgroup. **(B)** Analysis results of the top 15 most frequently reported PTs for each age subgroup. PT, preferred term.

To investigate gender-based differences in the safety signals of regorafenib, the ROR algorithm was utilized to screen regorafenib-related AEs separately for male and female patients. Analysis identified 148 AEs in males (Supplementary Table S4) and 137 AEs in females (Supplementary Table S5). Comparative analysis revealed that 83 AEs were observed in both genders, while distinct gender-specific AEs were identified. Among the shared AEs, gait inability, oral pain, mucosal inflammation, disseminated intravascular coagulation, decreased blood magnesium, and oral mucosal blistering demonstrated stronger associations with females. Conversely, palmar-plantar erythrodysaesthesia syndrome, decreased appetite, dysphonia, ascites, and chromaturia exhibited stronger associations with males (Figure 4A). The five most frequently reported AEs exclusive to females were pyrexia, hospitalisation, decreased platelet count, erythematous rash, and liver disorder. The five most frequently reported AEs exclusive to males were diarrhoea, upper abdominal pain, gait disturbance, dry skin, and general physical health deterioration.

Four statistical methods were employed to screen positive PTs within the age groups under 18 years, 18–65 years, and 65 years or older. These PTs were ranked by report frequency, with the top 15 presented for each subgroup. Distinct differences in AEs were observed across age groups, although palmar-plantar erythrodysaesthesia syndrome, fatigue, decreased appetite, asthenia, blister, and blood pressure increased were common symptoms present in all cohorts (Figure 4B).

### 3.4 Onset time of events

**FIGURE 5:**
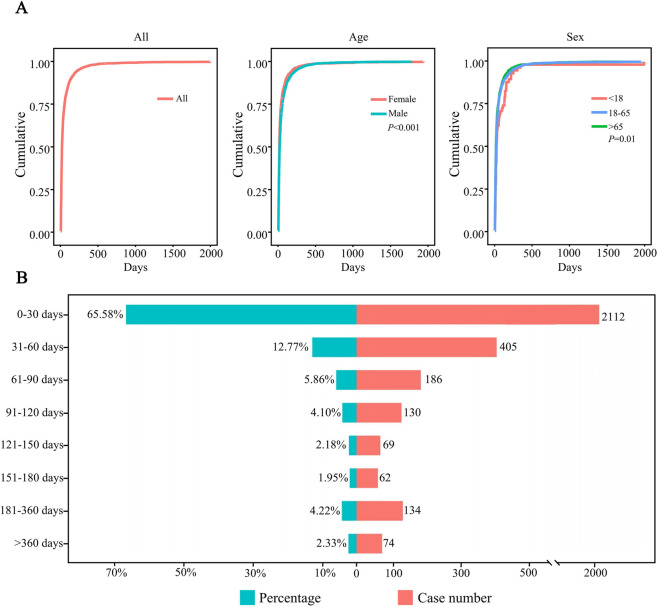
Time-to-event (TTO) analysis of patients treated with regorafenib. **(A)** Cumulative incidence of patients stratified by age and sex. **(B)** Number and proportion of patients stratified by time to onset.

After excluding patients with missing data, a total of 3,172 patients were included, and the median TTO of AEs was 15 days. To investigate the factors related to TTO, patients were stratified by age and sex. The median TTO in the <18, 18–65 and >65 years age groups were 22.5, 15 and 14, respectively (log-rank test, P = 0.01). Males had a median TTO of 18 days compared to 14 days for females (log-rank test, P < 0.001) (Table 4). The cumulative distribution curves illustrated the occurrence timeline of AEs in the whole and in different subgroups (Figure 5A). The number and proportion of patients stratified by TTO are shown in Figure 5B. The results indicated that the majority of regorafenib-associated AEs occurred within the initial 30-day period following treatment initiation.

**TABLE 4:** Time-to-onset analysis of patients stratified by age and sex.

Characteristic	All	Age			Sex	
	-	<18	18–65	>65	Male	Female
Min TTO (days)	1	2	1	1	1	1
Max TTO (days)	1920	1920	1887	1763	1763	1920
Median TTO (days)	15	22.5	15	14	18	14
Log-rank_test	-	*P* = 0.01			*P* < 0.001	

TTO, time to onset, N, number of patients with available TTO.

The Weibull distribution test for TTO indicated that the upper limit of the 95% CI for the shape parameter (β) was less than 1 in the overall population and in each subgroup, indicating an early failure type, suggesting that the probability of AEs gradually decreased over time (Table 5).

**TABLE 5:** Willbull distributions of patients stratified by age and sex.

Group	Sub group	Median (IQR) (days)	Weibull distribution				Failure type
			Shape parameter		Scale parameter		
			α	95% CI	β	95% CI	
All	​	15 (7–49)	37.64	35.56–39.72	0.67	0.65–0.68	Early failure
Age (years)	<18	22.5 (10–118)	61.18	35.48–86.89	0.65	0.53–0.77	Early failure
	18–65	15 (7–46)	37.39	34.22–40.56	0.67	0.64–0.69	Early failure
	>65	14 (7–44.25)	34.01	31.28–36.74	0.68	0.66–0.70	Early failure
Sex	Male	18 (7–55)	40.70	37.82–43.58	0.67	0.63–0.70	Early failure
	Female	14 (7–38)	32.37	29.45–35.29	0.66	0.63–0.69	Early failure

IQR, interquartile range; CI, confidence interval.

### 3.5 Clinical prioritization of relevant disproportionality signals

We performed a semiquantitative assessment of the clinical prioritization of 199 PTs that met all four algorithms. The majority of PTs (n = 103, 51.76%) were classified as low clinical priority, whereas 96 PTs demonstrated moderate to high clinical priority. Among the latter, hepatic failure (DME) and hepatitis fulminant (DME) constituted high-priority conditions, as presented in Supplementary Table S3, emphasizing the risk of liver failure during regorafenib treatment.

### 3.6 Analysis of factors influencing target PTs

To identify independent risk factors for hepatic failure and fulminant hepatitis, which were designated as our target PTs, a multivariable logistic regression model was applied to assess the various factors on target PTs, with considerations given to age, body weight and gender (Table 6). Key results indicated that elderly and male patients had a significantly higher likelihood of experiencing the target PTs, with ORs of 3.20 (95% CI: 1.49–7.37, P = 0.004) and 1.85 (95% CI: 1.47–2.33, P < 0.001). Additionally, the likelihood of target PT was significantly lower in the 80–100 kg group and the >100 kg group compared to the low-weight group (<80 kg), with ORs of 0.21 (95% CI: 0.13–0.32, P < 0.001) and 0.21 (95% CI: 0.09–0.41, P < 0.001).

**TABLE 6:** Multivariable logistic regression analysis of the odds ratio for target PTs controlling for demographic factors.

Predictor variables	Estimate	Std. error	z value	OR (95% CI)	P-value
(Intercept)	−2.80	0.33	−8.39	-	-
Age (18–65 years)	−0.02	0.34	−0.05	0.98 (0.54–2.02)	0.957
Age (≥65 years)	1.16	0.40	2.87	3.20 (1.49–7.37)	0.004
Weight (80–100 kg)	−1.55	0.23	−6.77	0.21 (0.13–0.32)	<0.001
Weight (>100 kg)	−1.56	0.39	−4.04	0.21 (0.09–0.41)	<0.001
Male	0.61	0.12	5.24	1.85 (1.47–2.33)	<0.001

The analysis adjusts for age (reference: <18 years), weight (reference: <80 kg), gender (reference: female).

OR, odds ratio; CI, confidential interval.

### 3.7 Targets network and protein-protein interactions

**FIGURE 6:**
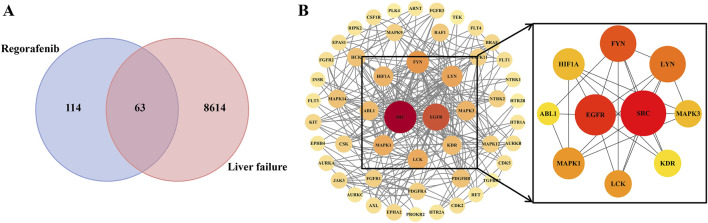
The protein–protein interaction network (PPI) of potential targets **(A)** and core targets **(B)**.

A total of 114 regorafenib target genes and 8,614 liver failure-related target genes were identified. Upon integration, 63 overlapping targets were identified, acting as potential candidates for regorafenib-induced liver failure (Figure 6A).

The PPI network comprised 51 nodes and 262 edges. From the network analysis, a set of 10 core targets was identified based on degree centrality, as illustrated in Figure 6B and depicted in Supplementary Table S6, suggesting that these genes may serve as key regulatory hubs in the pathogenesis of regorafenib-induced liver failure.

### 3.8 Gene ontology and pathway enrichment of overlapping genes

**FIGURE 7:**
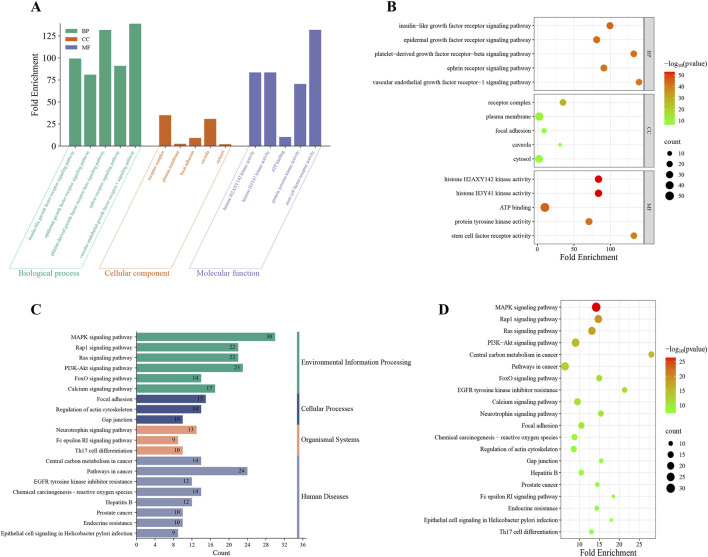
GO and KEGG enrichment analysis. The histogram **(A)** and the bubble chart **(B)** illustrated the top 5 enriched terms for each GO category (BP, CC and MF) on the 268 potential targets for regorafenib-induced liver failure. The histogram **(C)** and the bubble **(D)** illustrated the top 20 enriched KEGG pathways on 121 potential targets for regorafenib-induced liver failure.

GO enrichment analysis of the 63 overlapping targets identified 268 statistically significant GO entries: 148 biological processes (BP), 34 cellular components (CC), and 86 molecular functions (MF). The top 5 terms from each category, ranked by ascending false discovery rate (FDR), were selected for visualization (Figures 7A,B). KEGG pathway enrichment analysis revealed 121 significantly enriched pathways, with the top 20 (ranked by ascending FDR) visualized (Figures 7C,D).

Notably, GO analysis indicates these genes exhibit ubiquitous localization across subcellular structures. They are critically involved in signaling mechanisms initiated by specific growth factors or factor families (e.g., insulin-like growth factor, epidermal growth factor, platelet-derived growth factor, vascular endothelial growth factor) binding to cell membrane receptor tyrosine kinases, driving core cellular behaviors. KEGG analysis identified significant associations with regorafenib-related liver failure for multiple pathways, including the MAPK signaling pathway, Rap1 signaling pathway, PI3K-Akt signaling pathway, and Pathways in cancer. These pathways collectively form a core signaling network regulating fundamental cellular processes. Their significant dysregulation suggests a pivotal role in the onset and progression of regorafenib-induced liver failure.

### 3.9 Molecular docking for regorafenib and core target proteins

**FIGURE 8:**
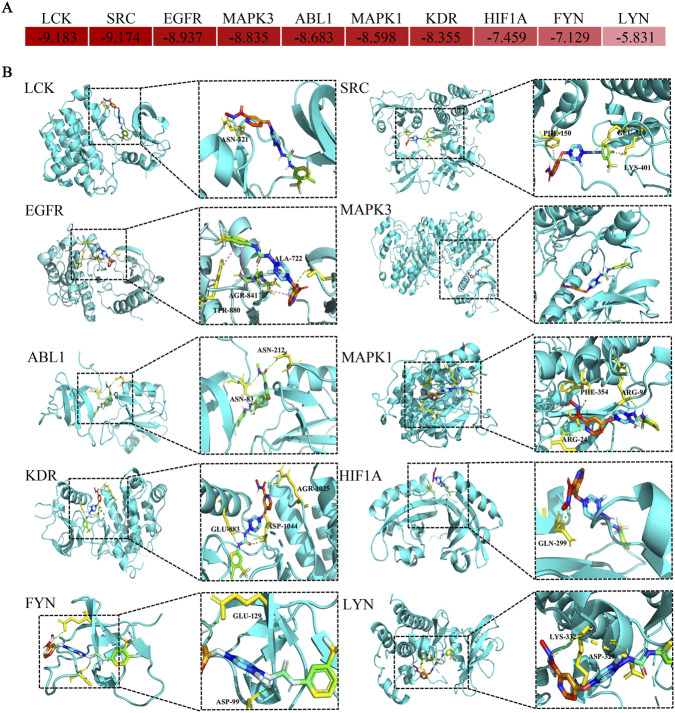
Diagram of molecular docking patterns between regorafenib and core targets. **(A)** Heat map of binding energy between regorafenib and proteins expressed by core toxicity genes. **(B)** Binding site of regorafenib to proteins expressed by core toxicity genes.

The docking simulation revealed robust interactions between regorafenib and the identified core target proteins. Specifically, the calculated binding free energies for all key complexes were consistently below −5 kcal/mol (Figure 8A), indicating that regorafenib can form thermodynamically stable bindings with these targets. Furthermore, the visualization of the binding modes demonstrated that regorafenib successfully anchored into the active pockets of the proteins through various molecular interactions (Figure 8B).

## 4 Discussion

Previous investigations into the safety of regorafenib have largely focused on data derived from clinical trials or standard descriptive pharmacovigilance analyses with smaller cohorts. While these studies established a baseline safety profile, they often lack the statistical power to comprehensively characterize rare, severe outcomes. This study advances the current understanding of regorafenib hepatotoxicity by leveraging a significantly larger sample size compared to prior works (Sakaeda et al., 2013). By utilizing this extensive real-world dataset, we were able to perform a robust multivariate analysis that identified higher body weight as a critical protective factor against liver failure—a nuance likely missed in smaller studies. Furthermore, we integrated this large-scale data mining with network toxicology and molecular docking, providing a unique translational link between statistical signals and their potential molecular drivers, specifically the dysregulation of MAPK and PI3K-Akt pathways.

We observed that regorafenib-related AE reports peaked in 2018 (n = 1,007) and subsequently declined. We speculate that this change may be related to the approval of the new-generation multi-targeted tyrosine kinase inhibitor lenvatinib for the treatment of hepatocellular carcinoma, which is also one of the main indications for regorafenib (Al-Salama et al., 2019). Lenvatinib became another option for patients after demonstrating noninferiority to sorafenib for its primary end point of overall survival (OS) but superiority for its secondary end points of progression-free survival (PFS) and objective response (Kudo et al., 2018), and patients in the lenvatinib group were more likely to discontinue treatment due to AEs (Gordan et al., 2024). Based on the information from the baseline profile, it was observed that the AEs of regorafenib occurred more commonly in males (54.67%) than in females (38.02%), due to the epidemiologic factors for major indications (Alvarez et al., 2024; Murphy and Zaki, 2024; Singal et al., 2023).

Our results confirm that regorafenib is associated with a spectrum of AEs predominantly affecting Hepatobiliary Disorders, Metabolism and Nutrition Disorders, and Neoplasms Benign, Malignant and Unspecified (Incl Cysts and Polyps) at the SOC level. Notably, Hepatobiliary Disorders emerged as the strongest signal, aligning with prior clinical trials such as the RESORCE ans CORRECT study, where liver-related toxicities were prominent (Finn et al., 2018; Grothey et al., 2013). This underscores the regorafenib’s hepatotoxic potential, with hepatic failure and fulminant hepatitis identified as high-priority designated medical events (DMEs), scoring maximally in our semi-quantitative clinical prioritization framework (Table 4). The mechanism of regorafenib-induced acute liver injury remains unclear. Previous studies have suggested that TKI hepatotoxicity is related to the effects of their metabolites on hepatic cell function (Teng et al., 2010). Some scholars have also proposed that liver injury may result from drug-induced hepatic blood flow impairment or sinusoidal obstruction syndrome (Akamine et al., 2015; Takahashi et al., 2016). A case report described regorafenib-associated autoimmune hepatitis that resolved after administration of azathioprine and prednisolone, suggesting a potential autoimmune mechanism (Kuwayama et al., 2016).

Palmar-plantar erythrodysesthesia (PPES), or hand-foot syndrome (HFS), is a common, dose-dependent toxicity associated with regorafenib and represents the costliest cutaneous adverse event to manage (Borovicka et al., 2011). Unlike chemotherapy-induced skin damage, multikinase inhibitor-related PPES predominantly affects pressure-bearing areas (palmar and plantar regions) rather than dorsal surfaces and is typically more localized (Robert et al., 2009). Consistent with prior reports, regorafenib-associated PPES frequently manifests early in therapy (median onset: 15 days), contrasting with the later onset (median: 72–79 days) observed with capecitabine (Azad et al., 2009). While the precise mechanism underlying this toxicity remains incompletely understood, it is hypothesized that regorafenib may disrupt the natural balance between vascular and epidermal injury and repair through effects on multiple molecular signaling pathways (Azad et al., 2009). Notably, PPES development correlates with prolonged overall and progression-free survival in patients receiving other multikinase inhibitors, suggesting a potential association with treatment efficacy (Uekusa et al., 2024). While rarely life-threatening, the high incidence of regorafenib-induced PPES in clinical practice necessitates prompt recognition and management.

Comparative analysis with existing literature reveals both consistencies and novel insights. For instance, common AEs like PPES, fatigue, and decreased appetite were prevalent across age groups, corroborating phase III trials where PPES affected up to 47% of patients (Grothey et al., 2013). However, our disproportionality analysis highlighted gender-specific differences: females showed stronger associations with mucosal inflammation and disseminated intravascular coagulation, while males were more prone to PPES and dysphonia (Figure 5A). This sexual dimorphism may stem from pharmacokinetic variations, such as differences in drug metabolism via cytochrome P450 enzymes, where females often exhibit higher plasma levels due to lower body weight and hormonal influences. Furthermore, body composition differences are statistically relevant; females generally have a higher percentage of body fat. Since regorafenib is a lipophilic drug with a large volume of distribution, gender-specific differences in adipose tissue distribution may alter drug accumulation and elimination half-lives. Age-related disparities were also evident; elderly patients (≥65 years) had a shorter median TTO (14 days) and higher odds of target PTs, possibly due to reduced hepatic reserve and polypharmacy. These subgroup analyses extend beyond previous reports, which often overlooked weight as a factor; our logistic regression revealed that higher body weight (>80 kg) significantly reduced the reporting odds of hepatic PTs, likely attributable to dose-independent pharmacokinetics (Keunecke et al., 2020). Clinically, since regorafenib is administered at a fixed dose (160 mg) rather than weight-based dosing, patients with higher body weight have lower systemic exposure (AUC) per kilogram compared to lighter patients. This suggests that the standard fixed dose may lead to supratherapeutic concentrations in patients with low body weight (<50 kg), warranting consideration for dose reduction in this specific subgroup. This suggests that implementing individualized dose adjustments based on weight and gender in clinical practice may effectively reduce the risk of toxicity.

The temporal profile of AEs, characterized by an early failure type in Weibull distribution (β upper 95% CI < 1), indicates that most events (66.58% within 30 days) occur shortly after initiation, consistent with regorafenib’s rapid onset of kinase inhibition. This pattern implies a need for intensive monitoring in the first month, particularly for high-priority signals like hepatic failure, which carried a case fatality rate warranting urgent intervention. Based on these findings, we propose a risk-adapted monitoring strategy. While standard labeling suggests monitoring liver function tests (ALT, AST, bilirubin) every two weeks during the first two months, our data supports a more aggressive approach for high-risk populations identified in this study—specifically males and elderly patients (≥65 years). For these groups, we recommend weekly liver function monitoring during the first cycle (28 days) to ensure early detection of hepatotoxicity before it progresses to irreversible failure. Prompt recognition during this critical 30-day window allows for timely dose interruption or reduction, which is vital given the rapid onset of kinase inhibition.

To uncover the molecular mechanisms, we employed network toxicology and identified 63 candidate targets bridging regorafenib and liver failure (Figure 6A). This overlap underscores the polypharmacological nature of regorafenib, a multi-kinase inhibitor, which may inadvertently disrupt hepatic homeostasis through off-target effects (Kabir and Muth, 2022).

To further delineate interactions among these targets, a PPI network was constructed. Topology analysis highlighted 10 core targets (Figure 6B) serving as pivotal regulatory hubs in the pathogenesis of liver failure. These core targets, including key signaling nodes, highlight regorafenib’s interference with interconnected protein modules that govern cellular responses to stress, proliferation, and apoptosis in hepatocytes.

PPI network analysis identified the main targets of liver failure in the treatment of regorafenib; the molecular docking and molecular dynamics simulation results also exhibited good binding affinity and stability. Although this study lacks direct experimental validation, these findings are consistent with the known pharmacological profile of regorafenib. Besides, KEGG pathway enrichment analysis showed that MAPK and PI3K-AKT signal pathways were significantly enriched. Existing studies have demonstrated that excessive activation of the MAPK pathway can exacerbate liver failure, whereas physiological levels of activation promote liver regeneration (Ni et al., 2024). Activation of the PI3K-Akt pathway exerts a protective effect on hepatocytes, significantly reducing markers of liver injury and cell death while mitigating oxidative damage in the context of hepatic ischemia-reperfusion injury (Li et al., 2023; Shamsan et al., 2024). As a multi-targeted tyrosine kinase inhibitor, regorafenib can inhibit the activation of these two pathways through direct or indirect mechanisms, thereby enhancing its anti-tumor effects while potentially promoting liver failure. Meanwhile, the MAPK pathway can negatively regulate the PI3K-Akt pathway in hepatocytes, and dual targeting of these pathways (e.g., inhibiting MAPK while activating PI3K-Akt) is regarded as a promising therapeutic strategy (Gan et al., 2025). Therefore, while our molecular docking results confirm a strong binding affinity between regorafenib and these targets, future *in vitro* and *in vivo* studies are warranted to definitively verify whether the blockade of MAPK and PI3K-Akt signaling is the direct cause of the observed hepatic failure.

This study also has some shortcomings and limitations: (1) As a spontaneous reporting system, FAERS may suffer from omissions, duplications, incomplete reports, and misreporting, which can introduce various forms of reporting bias (e.g., under-reporting, over-reporting, confounding by indication) due to the varying knowledge backgrounds of reporters (Alomar et al., 2020). Therefore, the signal strengths (ROR/PRR) represent statistical associations within the database, not absolute clinical risk (Xia et al., 2023). (2) While we utilized multivariable logistic regression to control for demographic factors (age, gender, weight), we could not account for unobserved confounders such as comorbidities, genetic susceptibility, and all concomitant medications due to incomplete data entries. Consequently, the identified factors should be interpreted as being associated with a higher likelihood of reporting, rather than confirmed independent risk factors for liver failure. Causality cannot be definitively established based solely on this retrospective analysis (Crisafulli et al., 2023). (3) In network toxicology, target prediction for regorafenib heavily relies on database algorithms. While molecular docking showed strong binding affinities and literature comparison supports the involvement of MAPK/PI3K-Akt pathways, these findings lack direct ‘wet-lab’ experimental validation (e.g., Western blot or PCR) in this study. Future *in vivo* and *in vitro* experiments are necessary to confirm the precise regulatory roles of these targets in regorafenib-induced hepatotoxicity (Liao et al., 2022).

In conclusion, our analysis of the FAERS database characterizes the real-world safety profile of regorafenib, highlighting severe liver injury as a priority safety signal that frequently occurs early in treatment. We identified male sex and advanced age as significant risk factors, whereas higher body weight appeared protective. Furthermore, network toxicology predictions suggest that the MAPK and PI3K-Akt signaling pathways may mediate these adverse effects. These findings underscore the importance of early hepatic function monitoring, particularly in high-risk populations, to mitigate drug-induced liver injury.

## Data Availability

The original contributions presented in the study are included in the article/Supplementary Material, further inquiries can be directed to the corresponding authors.
