# Supplementary material for: Real-world safety profile and mechanistic insights into regorafenib-induced liver failure: a pharmacovigilance study integrated with network toxicology
Source: Front Pharmacol. 2026 Jan 12;16:1698511. doi: 10.3389/fphar.2025.1698511 (PMC12832792; doi:10.3389/fphar.2025.1698511)
Supplement: Supplementary file 3 [file Table1.docx]

**Supplementary Table S1** A two-by-two contingency table and detailed formulas for disproportionality analysis.

|  | Target adverse drug events | Other adverse drug events | Sums |
| --- | --- | --- | --- |
| Regorafenib | a | b | a+b |
| Other drugs | c | d | c+d |
| Sums | a+c | b+d | a+b+c+d |

| Algorithms | Equation | Criteria |
| --- | --- | --- |
| ROR | ROR=ad/b/c | lower limit of 95% CI>1, N≥3 |
|  | 95%CI=e^ln(ROR)±1.96(1/a+1/b+1/c+1/d)^0.5^ |  |
| PRR | PRR=a(c+d)/c/(a+b) | PRR≥2, χ^2^≥4, N≥3 |
|  | χ^2^=[(ad-bc)^2](a+b+c+d)/[(a+b)(c+d)(a+c)(b+d)] |  |
| BCPNN | IC=log_2_a(a+b+c+d)(a+c)(a+b) | IC025>0 |
|  | 95%CI= E(IC) ± 2V(IC)^0.5 |  |
| MGPS | EBGM=a(a+b+c+d)/(a+c)/(a+b) | EBGM05>2 |
|  | 95%CI=e^ln(EBGM)±1.96(1/a+1/b+1/c+1/d)^0.5^ |  |

Equation: a, number of reports containing both the target drug and target adverse drug reaction; b, number of reports containing other adverse drug reaction of the target drug; c, number of reports containing the target adverse drug reaction of other drugs; d, number of reports containing other drugs and other adverse drug reactions. 95%CI, 95% confidence interval; N, the number of reports; χ^2^, chi-squared; IC, information component; IC025, the lower limit of 95% CI of the IC; E(IC), the IC expectations; V(IC), the variance of IC; EBGM, empirical Bayesian geometric mean; EBGM05, the lower limit of 95% CI of EBGM.
