# Supplementary material for: Real-world safety profile and mechanistic insights into regorafenib-induced liver failure: a pharmacovigilance study integrated with network toxicology
Source: Front Pharmacol. 2026 Jan 12;16:1698511. doi: 10.3389/fphar.2025.1698511 (PMC12832792; doi:10.3389/fphar.2025.1698511)
Supplement: Supplementary file 5 [file Table6.docx]

**Supplementary Table S6** Core targets screened from the PPI network.

| **Name** | **Degree** | **Closeness centrality** | **Betweenness centrality** | **Topological coefficient** |
| --- | --- | --- | --- | --- |
| **SRC** | 56 | 0.568181818 | 0.444104927 | 0.155357143 |
| **EGFR** | 40 | 0.526315789 | 0.201674677 | 0.175 |
| **FYN** | 26 | 0.462962963 | 0.064551698 | 0.236538462 |
| **LYN** | 22 | 0.420168067 | 0.021205112 | 0.293506494 |
| **MAPK1** | 20 | 0.442477876 | 0.03245484 | 0.245945946 |
| **LCK** | 20 | 0.41322314 | 0.028943693 | 0.285294118 |
| **HIF1A** | 18 | 0.45045045 | 0.200486085 | 0.255255255 |
| **MAPK3** | 18 | 0.427350427 | 0.016012987 | 0.26984127 |
| **KDR** | 16 | 0.431034483 | 0.060426098 | 0.288194444 |
| **ABL1** | 16 | 0.406504065 | 0.055012987 | 0.25 |

PPI, protein–protein interaction network; SRC, SRC Proto-Oncogene; EGFR, Epidermal Growth Factor Receptor; FYN, FYN Proto-Oncogene; LYN, LYN Proto-Oncogene; MAPK1, Mitogen-Activated Protein Kinase 1; LCK, LCK Proto-Oncogene; HIF1A, Hypoxia-Inducible Factor 1-Alpha; MAPK3, Mitogen-Activated Protein Kinase 3; KDR, Kinase Insert Domain Receptor; ABL1 ,ABL Proto-Oncogene 1.
